# COL9A1 Gene Polymorphism Is Associated with Kashin-Beck Disease in a Northwest Chinese Han Population

**DOI:** 10.1371/journal.pone.0120365

**Published:** 2015-03-16

**Authors:** Xiaowei Shi, Feng Zhang, Aili Lv, Yan Wen, Xiong Guo

**Affiliations:** 1 Center of Maternal and Child Health Care, The First Affiliated Hospital of Medical Collage of Xi’an Jiaotong University, Xi’an, Shannxi, PR of China; 2 School of Public Health, Health Science Center, Xi’an Jiaotong University, Key Laboratory of Environment and Gene Related Diseases of Ministry of Education, Key Laboratory of Trace Elements and Endemic Diseases of Ministry of Health, Xi’an, Shannxi, PR of China; University of Texas Health Science Center at San Antonio, UNITED STATES

## Abstract

**Objective:**

We sought to determine whether genomic polymorphism in collagen IX genes (COL9A) was associated with Kashin-Beck disease (KBD).

**Methods:**

Twenty seven single nucleotide polymorphisms (SNPs) in COL9AI, COL9A2 and COL9A3 were genotyped in 274 KBD cases and 248 healthy controls using the Sequenom MassARRAY system. Associations between the COL9A polymorphism and KBD risk were detected using an unconditional logistic regression model. Linkage disequilibrium (LD) and haplotypes analysis were performed with the Haploview software.

**Results:**

After Bonferroni correction, the frequency distribution of genotypes in rs6910140 in COL9A1 was significantly different between the KBD and the control groups (*X* ^2^ = 16.74, *df* = 2, *P* = 0.0002). Regression analysis showed that the allele “C” in SNP rs6910140 had a significant protective effect on KBD [odds ratio (OR) = 0.49, 95% confidence interval (CI) = 0.34–0.70, P = 0.0001]. The frequencies of alleles and genotypes in rs6910140 were significantly different among subjects of different KBD stages (allele: *X* ^2^ = 7.82, *df* = 2, *P* = 0.02, genotype: *X* ^2^ = 14.81, *df* = 4, *P* = 0.005). However, haplotype analysis did not detect any significant association between KBD and COL9A1, COL9A2 and COL9A3.

**Conclusions:**

We observed a significant association between rs6910140 of COL9A1 and KBD, suggesting a role of COL9A1 in the development of KBD.

## Introduction

Kashin-Beck disease (KBD) is a chronic osteochondropathy affecting the bones and joints that is endemic to certain geographical areas. A key pathological feature of KBD is chondrocyte necrosis in the deep zone of the growth plate of cartilage and articular cartilage [1],[2]. Clinically, the disease usually presents in childhood, between 5 and 13 years of age, and mainly attacks the growth plate cartilage. KBD presents as dwarfism, very short upper limbs, and deformed and painful joints.

The etiology of KBD remains unclear. In the past 150 years, three environmental hypotheses have been proposed: selenium deficiency, mycotoxins from contaminated storage grains, and organic matter (e.g., fulvic acid or FA) in drinking water [3–5]. Recent epidemiological and genetic study results also suggest the interaction between environment factors and susceptibility genes might play a role in the disease[6],[7]. Certain susceptibility genes may affect susceptibility to environmental factors, such as selenium deficiency or other biologic factors [8–11].

Type IX collagen, a trimer of three different gene products, a1(IX), a2 (IX) and a3 (IX) chains, are encoded by the COL9A1, COL9A2, and COL9A3 genes, is quantitatively a minor component that functions structurally by covalently cross-linking to the surface of type II collagen fibrils [12]. The skeletal consequences of mutations in collagen IX genes in humans and animals strongly suggest that the proteinmolecule is essential for the functional longevity of joint cartilages and connected with osteochondropathy[13],[14]. Mutations in collagen IX genes have been shown to cause multiple epiphyseal dysplasia (MED) in adult patients [15]. In addition, a suggestive linkage has been reported between COL9A1 and hip osteoarthritis (OA) in female patients [16]. Further support for the possible role of collagen IX in OA has been obtained from animal studies, mice lacking the a1(IX) chains developed degenerative joint disease similar to human OA[17]. KBD has a common pathological feature, arthritic hyaline cartilage damage, with OA [7]. However, few studies have identified regions of the genome that contain genes predisposed to KBD. In this study, we evaluate for the first time the impact of genomic polymorphism of COL9A on the risk and progression of KBD.

## Materials and Methods

### Study population

In total, 522 unrelated Chinese Han individuals were included in this study. These individuals were collected from KBD-endemic areas of the Linyou and Yongshou counties of Shaanxi province, in northwest China. This group consisted of 274 KBD patients and 248 healthy controls. Radiographs of the right hand were taken for both the KBD patients and the healthy controls and read by veteran orthopedists. KBD was diagnosed according to the national diagnostic criteria of China (WS/T 207–2010). Patients with clinical symptoms or radiographic changes of other osteochondropathy were excluded. The healthy control was defined as no KBD and OA. The controls were randomly selected and were frequency-matched by age (53.37±10.79 years vs 51.71±17.85 years, *t* = 1.29, *P*>0.05) and sex (male/female, 125/149 vs 124/124, *x* ^*2*^ = 1, *P*>0.05) (Table 1), and cases were excluded if his/her first to third-degree relative had been selected. Fresh blood (5 mL) was collected from each subject. The study was performed in accordance with the Declaration of Helsinki and approved by the Human Ethics Committee of Xi'an Jiaotong University, PR of China. Written informed consent was also obtained from the subjects or their relatives.

**Table 1:** The characteristics of study subjects by groups.

Characteristics	Cases (n = 274)	Controls (n = 248)	P values
Gender, male/ female	125/149	124/124	0.32
Age, Mean±SD (year)	53.37±10.79	51.71±17.85	0.19
KBD stages			
I	158	0	/
II	85	0	/
III	31	0	/

### SNP Selection

Based on the information of SNPs in COL9A that predispose for OA [18–20]. 27 SNPs (15 SNPs in COL9A1, 8 SNPs in COL9A2, 4 SNPs in COL9A3) were selected from the NCBI SNP and HapMap database, and evaluated in this study. The selected SNPs were required to have a minor allele frequency (MAF) ≥5% [21]. The information of 27 SNPs was shown in Table 2.

**Table 2:** The loci information of the 27 SNPs in COL9A1、COL9A2 and COL9A3 genes.

Gene and SNP	Chromosome position	Alleles ^a^	SNP location	MAF	HEW test (*P*)
**COL9A1**					
rs592121	6:71041157	A/G	exon	0.292	0.21
rs1064250	6:70983055	A/G	3'UTR	0.292	0.89
rs1135056	6:71018554	T/C	exon	0.286	0.41
rs2274584	6:71018760	C/T	Intron	0.127	0.14
rs3806099	6:71046424	A/G	Intron	0.292	0.24
rs519068	6:71018765	T/C	Intron	0.286	0.88
rs617600	6:71045633	A/C	Intron	0.133	0.52
rs617985	6:71045670	C/T	Intron	0.133	0.52
rs679521	6:71048909	G/C	Intron	0.489	0.61
rs6928611	6:71047486	G/C	Intron	0.067	0.18
rs2072650	6:71001006	C/T	exon	0.071	0.93
rs12210870	6:71027222	A/G	Intron	0.060	0.55
rs6910140	6:71000978	T/C	exon	0.149	0.73
rs883708	6:71033259	A/G	Intron	0.078	0.75
rs616642	6:71045407	A/G	Intron	0.072	0.22
**COL9A2**					
rs12077871	1:40545737	G/A	exon	0.101	0.61
rs2076696	1:40544560	C/G	Intron	0.304	0.95
rs209923	1:40557522	A/C	Intron	0.367	0.63
rs1983658	1:40778377	T/C	Intron	0.300	0.68
rs3737821	1:40540931	G/A	exon	0.059	0.88
rs2075560	1:40545876	C/T	Intron	0.163	0.95
rs2273195	1:40552428	G/T	Intron	0.169	0.83
rs2228567	1:40773123	G/C	Intron	0.085	0.17
**COL9A3**					
rs2249766	20:60928992	T/A	Intron	0.289	0.44
rs3765462	20:60938146	G/A	Intron	0.125	0.21
rs741758	20:60933946	T/C	Intron	0.101	0.27
rs760087	20:60938245	A/G	Intron	0.429	0.27

^a^ Stands for the major/minor alleles

### Genotyping analysis

Genomic DNA was extracted from the peripheral blood of the 274 KBD patients and 248 healthy controls using a blood DNA extraction kit (TIANGEN, Beijing, China). Genotyping was performed using the Sequenom MassARRAY system. Primers were designed using Sequenom SNP Assay Design software version 3.0 for iPLEX reactions. The PCR contained: 0.8 μL H_2_O, 0.5 μL PCR buffer (20 mM MgCl_2_), 0.4 μL 25 mM MgCl_2_, 0.1 μL 25 mM dNTP mix, 1 μL primer mix (500 nM each), 0.5 μL QC competitor, 0.5 μL QA spike, 0.2 μL PCR enzyme and 1 μL sample DNA. The PCR was performed in a GeneAmp PCR System 9700 thermal cycler (ABI 9700, PerkinElmer, U.S.A.) under the following conditions: denaturation at 95°C for 2 min, 45 cycles of 95°C for 30 s, 56°C for 30 s, and 72°C for 1 min, and extension at 72°C for 5 min. The PCR products were treated with a cocktail of 1.53 μL H_2_O, 0.17 μL 10×shrimp alkaline phosphatase (SAP) buffer and 0.3 μL SAP (1.7 U/m L) in a GeneAmp PCR System 9700 thermal cycler at 37°C for 40 min followed by 85°C for 5 min. The sing base extension (SBE) reaction contained 7 μL of the SAP-treated PCR products and 2 μL of iPLEX Pro mix (Sequenom). The iPLEX Pro mix contained 0.2 μL 10×iPLEX Pro buffer, 0.2 μL iPLEX Pro termination mix, 0.94 μL primer mix (0.74–1.46 μM, Sequenom), 0.041 μL iPLEX1 enzyme, 0.5 μL EXT QA spike, and 0.119 μL H_2_O. The SBE reaction was performed in a GeneAmp PCR System 9700 thermal cycler under the following conditions: denaturation at 94°C for 30 s, 40 cycles of 94°C for 5 s and five cycles of 52°C for 5 s and 80°C for 5 s, and extension at 72°C for 3 min. A total of 41 μL of molecular-grade H_2_O and ion exchange resin (Sequenom) was then added to each sample and the results were visualized using a MassARRAY Analyzer 4 system (Sequenom) using autorun settings.

### Statistical analysis

The Hardy-Weinberg equilibrium (HWE) of each SNP was tested by the goodness-of-fit *x* ^2^ test to compare the expected frequencies of genotypes in controls, SNPs with *P*>0.05 were considered to be in HWE [22]. The Independent-Samples T test was used to determine differences according to age, and the chisquare or Fisher's exact test was performed to calculate the clinical parametric distributions. Unconditional logistic regression analysis models were used to evaluate the relationships between different genotypes and disease risk [Odds ratios (OR), 95% confidence intervals (95% CI)] adjusted by age and gender [23,24].To account for multiple testing, Bonferroni correction was applied. Significant associations were defined at p value<0.05/27 = 0.0018. [24]. Haplotypes and haplotype frequencies were calculated using Haploview software (version 4.2). Haplotypes with frequency less than 1% were combined. Statistical analysis was carried out using SPSS 17.0 for Windows. The haplotype with p value<0.05 was considered statistically significant.

## Results

### Baseline characteristics

A total of 274 KBD patients and 248 age and sex matched controls were included in this study. No significant differences were observed between KBD and control group in age (53.37±10.79 vs 51.71±17.85, *t* = 1.29, *P*>0.05) and sex (male/female, 125/149 vs 124/124, *x* ^2^ = 1,*P*>0.05). In addition, the clinical stages of the 274 KBD patients were divided into I, II and III stages and the constituent ratio was 57.66%(158/274), 31.02%(85/274) and 11.32%(31/274) respectively (Table 1).

### Association of studied SNPs in genes COL9A1, COL9A2 and COL9A3 with KBD

A case-control comparison of both the genotype and allele frequencies for the 27 SNPs is presented in Table 3. All tested SNPs were in HWE (*X* ^2^ = 0.10–3.98, *df* = 2, *P* = 0.14–0.95) (Table 2). When the allele frequencies were compared between the KBD and controls, a significant *X* ^2^ value was detected at rs6910140 of COL9A1 gene (*X* ^2^ = 7.58, *df* = 1, *P = 0.005*). The distribution frequencies of genotypes in rs1135056 and rs6910140 (*X* ^2^ = 8.12, *df* = 2, *P* = 0.017; *X* ^2^ = 16.74, *df* = 2, *P* = 0.0002 respectively) were significantly different between the two groups (Table 3). However, the significant associations of allele frequency of rs6910140 and genotype frequency of rs1135056 did not survive Bonferroni correction (*P*>0.0018).

**Table 3:** Comparison of genotype and allele frequencies between cases and controls.

Gene/ SNP	Genotype (%) ^a^			Minor allele frequency (%)		
	KBD (N = 274)	Controls (N = 248)	P values	KBD	Controls	P values
**COL9A1**						
rs592121	44.5/45.6/9.9	45.6/46.4/8.1	0.76	32.7	31.3	0.61
rs1064250	60.4/36.6/2.9	63.0/32.5/4.4	0.46	21.2	20.7	0.82
rs1135056	47.8/37.6/14.6	47.6/45.2/7.2	**0.017**	33.3	29.8	0.22
rs2274584	60.6/33.6/5.8	65.1/29.3/5.6	0.55	22.6	20.3	0.33
rs3806099	43.9/45.4/10.7	45.8/46.1/8.1	0.59	33.3	31.2	0.45
rs519068	49.3/44.2/6.6	55.0/38.5/6.4	0.40	28.6	25.7	0.29
rs617600	74.4/24.9/0.7	74.6/23.0/2.4	0.26	13.2	13.9	0.73
rs617985	74.5/24.8/0.7	74.6/23.0/2.4	0.26	13.1	13.9	0.72
rs679521	29.3/49.5/21.2	27.8/48.4/23.8	0.41	45.9	48.0	0.54
rs6928611	84.3/15.0/0.7	81.1/17.7/1.2	0.08	8.2	10.0	0.29
rs2072650	75.5/23.8/0.7	79.1/19.7/1.2	0.46	12.6	11.0	0.43
rs12210870	88.7/10.9/0.4	90.3/8.9/0.8	0.21	5.8	5.2	0.67
rs6910140	70.4/27.7/1.8	53.6/41.5/4.8	**0.0002**	15.6	25.6	**0.005**
rs883708	73.8/24.7/1.5	75.3/22.7/2.0	0.78	13.8	13.3	0.82
rs616642	75.9/23.4/0.7	77.4/20.2/2.4	0.21	12.4	12.5	0.96
**COL9A2**						
rs12077871	78.5/20.7/0.7	76.2/22.6/1.2	0.75	11.1	12.5	0.49
rs2076696	53.6/40.5/5.8	59.1/35.6/5.3	0.45	26.1	23.1	0.26
rs209923	44.2/42.0/13.8	42.9/44.1/13.0	0.88	34.8	35.0	0.93
rs1983658	41.3/43.5/15.2	36.7/46.9/16.3	0.53	37.0	39.8	0.36
rs3737821	83.9/15.7/0.4	81.8/17.3/0.8	0.69	8.2	9.5	0.47
rs2075560	73.0/23.7/3.3	71.5/26.0/2.4	0.73	15.1	15.5	0.89
rs2273195	68.9/26.7/4.4	66.5/30.2/3.2	0.57	17.8	18.3	0.81
rs2228567	82.8/16.4/0.7	83.5/16.1/0.4	0.27	8.9	8.5	0.78
**COL9A3**						
rs2249766	51.7/40.2/8.1	59.3/34.3/6.5	0.21	28.2	23.6	0.09
rs3765462	69.6/28.9/1.5	75.5/21.7/2.8	0.11	15.9	13.7	0.30
rs741758	70.0/26.4/3.7	67.2/30.8/2.0	0.32	16.8	17.4	0.81
rs760087	38.4/43.9/17.7	35.2/51.0/13.8	0.22	39.7	39.3	0.89

a Homozygote of the major allele/ heterozygote /homozygote of the minor allele.

ORs and 95% CIs for KBD were calculated from unconditional logistic regression model for evaluating relative risks (Table 4). A weak association with increased KBD risk was observed among individuals with the heterozygous variant genotype (GA) at rs3765462 (adjusted OR:1.59, 95% CI: 1.05–2.42, *P* = 0.03), compared with the homozygous wild type (GG). Dominant model and recessive model were also applied for detecting association between COL9A and KBD. Adjusted ORs for rs1135056 in COL9A1 in the recessive model and rs6910140 in COL9A1 in the dominant model were statistically significant (OR = 2.13, 95% CI = 1.17–3.82, *P* = 0.01 and OR = 0.49, 95% CI = 0.34–0.70, *P* = 0.0001; respectively). But after Bonferroni correction, only the SNP rs6910140 in the dominant model remained. The allele “C” of rs6910140 in COL9A1 had a significant protective effect on KBD.

**Table 4:** Analysis of association of the 27 SNPs gene polymorphism with the risk of KBD ^a^.

Gene/SNP	Genotype model^b^			Dominant model	Recessive model	Allele model ^c^
	AA	AB	BB			
	OR (95% CI)	OR (95% CI)	OR	OR (95% CI)	OR (95% CI)	OR (95% CI)
**COL9A1**						
rs592121	1.05(0.54–2.02)	0.98 (0.67–1.43)	1.00	1.04(0.74–1.48)	1.26(0.69–2.30)	1.01(0.76–1.32)
rs1064250	0.60 (0.22–1.64)	1.19(0.82–1.75)	1.00	1.12(0.78–1.59)	1.20 (0.83–1.72)	1.03 (0.75–1.40)
rs1135056	0.73 (0.36–1.50)	1.25(0.86–1.82)	1.00	0.99(0.70–1.40)	**2.13(1.17–3.82)** ^d^	1.12 (0.85–1.46)
rs2274584	1.08 (0.50–2.34)	1.27(0.85–1.87)	1.00	1.21(0.85–1.73)	1.04 (0.50–2.18)	1.16 (0.85–1.57)
rs3806099	1.14 (0.59–2.18)	0.99(0.68–1.45)	1.00	1.08(0.76–1.52)	1.36 (0.75–2.47)	1.04 (0.78–1.36)
rs519068	0.94 (0.46–1.97)	1.19(0.82–1.73)	1.00	1.26(0.89–1.78)	1.02 (0.51–2.05)	1.07 (0.81–1.42)
rs617600	0.23 (0.04–1.21)	1.04(0.69–1.59)	1.00	1.01(0.68–1.50)	0.30 (0.06–1.49)	0.89 (0.61–1.28)
rs617985	0.23 (0.04–1.21)	1.04(0.68–1.58)	1.00	1.01(0.68–1.49)	0.30 (0.06–1.48)	0.88 (0.61–1.28)
rs679521	0.66 (0.39–1.09)	0.85 (0.56–1.32)	1.00	0.84(0.57–1.23)	0.78 (0.52–1.16)	0.81 (0.63–1.05)
rs6928611	0.00 (0.00-/)	0.83(0.51–1.34)	1.00	0.80(0.51–1.26)	0.87 (0.55–1.37)	0.73 (0.47–1.14)
rs2072650	0.56 (0.08–3.85)	1.25(0.81–1.93)	1.00	1.23(0.82–1.86)	0.61 (0.10–3.65)	1.15 (0.77–1.70)
rs12210870	0.00 (0.00-/)	1.55(0.83–2.92)	1.00	1.64(0.89–3.01)	1.58 (0.86–2.91)	1.60 (0.87–2.94)
rs6910140	0.48 (0.16–1.47)	0.71(0.47–1.05)	1.00	**0.49(0.34–0.70)** ^e^	0.37(0.13–1.05)	0.73 (0.52–1.01)
rs883708	0.59 (0.15–2.35)	1.07(0.70–1.64)	1.00	1.08 (0.73–1.61)	0.73 (0.19–2.73)	1.01 (0.68–1.43)
rs616642	0.23 (0.04–1.21)	1.03 (0.67–1.60)	1.00	1.09 (0.72–1.63)	0.30 (0.06–1.48)	0.87 (0.59–1.27)
**COL9A2**						
rs12077871	0.50 (0.08–3.09)	0.85(0.55–1.32)	1.00	0.88 (0.58–1.32)	0.61 (0.10–3.68)	0.83 (0.56–1.23)
rs2076696	1.56 (0.69–3.51)	1.22(0.84–1.77)	1.00	1.25 (0.88–1.77)	1.12 (0.53–2.37)	1.22 (0.91–1.64)
rs209923	0.94 (0.54–1.66)	0.86 (0.58–1.26)	1.00	0.95 (0.67–1.34)	1.07 (0.64–1.78)	0.94 (0.72–1.22)
rs1983658	0.71 (0.41–1.22)	0.76(0.51–1.14)	1.00	0.81 (0.57–1.16)	0.92 (0.57–1.48)	0.82 (0.63–1.07)
rs3737821	0.44 (0.03–5.57)	0.88(0.54–1.41)	1.00	0.86 (0.55–1.36)	0.45 (0.04–4.99)	0.85 (0.55–1.32)
rs2075560	1.34 (0.45–4.02)	0.86 (0.57–1.30)	1.00	0.93 (0.63–1.37)	1.36 (0.48–3.87)	0.96 (0.67–1.36)
rs2273195	1.28(0.48–3.39)	0.83 (0.55–1.23)	1.00	0.90 (0.62–1.30)	1.38 (0.55–3.43)	0.93 (0.67–1.29)
rs2228567	0.99 (0.00-/)	0.9 (0.61–1.12)	1.00	1.08 (0.68–1.72)	1.03 (0.64–1.64)	1.07 (0.68–1.69)
**COL9A3**						
rs2249766	1.58 (0.78–3.23)	1.37 (0.94–2.01)	1.00	1.36 (0.96–1.93)	1.28 (0.66–2.50)	1.32 (0.99–1.77)
rs3765462	0.77 (0.21–2.85)	**1.59(1.05–2.42)** ^d^	1.00	1.35 (0.91–1.98)	0.51 (0.15–1.78)	1.34 (0.94–1.92)
rs741758	1.36 (0.54–4.13)	0.81 (0.55–1.22)	1.00	0.88 (0.61–1.27)	1.84 (0.62–5.46)	0.92 (0.66–1.29)
rs760087	1.11 (0.64–1.91)	0.79 (0.54–1.81)	1.00	0.87 (0.61–1.25)	1.35 (0.84–2.17)	1.03 (0.77–1.29)

^a^ Adjusted for age and gender;

^b^ AB stands for the minor/major alleles, BB as the reference genotype;

^c^ Major allele as the reference allele;

^d^ The significance did not remain after correction for multiple testing;

^e^ The significance remained after correction for multiple testing.

**Fig 1:**
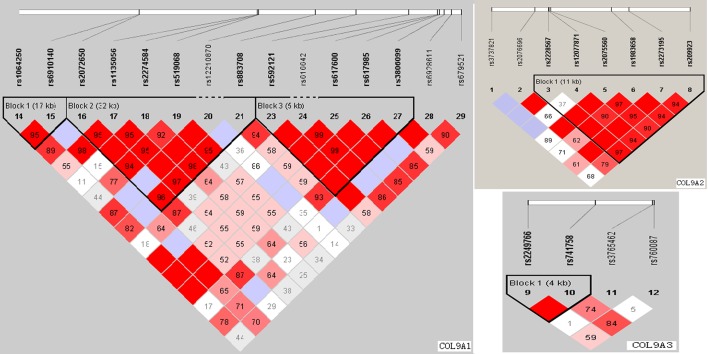
The haplotype blocks of the three studied genes: COL9A1, COL9A2 and COL9A3. The numbers indicate the extent of linkage disequilibrium based on D’ value between 2 SNPs calculated with Haploview 4.2

Linkage disequilibrium structure is shown in Fig. 1. COL9A1 included 3 haplotype blocks, whereas COL9A2 and COL9A3 had only 1. The haplotypes of the different blocks of each gene were calculated as shown in Table 5. The most frequent haplotype was used as reference, haplotype analysis of genes COL9A1、COL9A2 and COL9A3 did not detected any significant association with KBD (Table 5).

**Table 5:** Results of the haplotype analysis for the genes COL9A1、COL9A2 and COL9A3.

Gene	Block	Haplotype	Controls (freq.)	Cases (freq.)	OR (95% CI)	P value
COL9A1	Block 1	AT	0.7909	0.7835	1.00	---
		GT	0.1087	0.1381	1.28 (0.87–1.89)	0.21
		GC	0.0982	0.0742	0.76 (0.49–1.19)	0.23
	Block 2	CTCTA	0.7083	0.7006	1.00	---
		TCTCG	0.0977	0.1146	1.16 (0.77–1.76)	0.48
		CCTCA	0.0543	0.0820	1.53 (0.92–2.54)	0.10
		CCCCA	0.0698	0.0547	0.80 (0.48–1.34)	0.40
		CCTCG	0.0248	0.0166	0.61 (0.25–1.48)	0.27
		CCCTA	0.0149	0.0010	0.62 (0.19–2.01)	0.43
	Block 3	AACA	0.6867	0.6660	1.00	---
		GACG	0.1718	0.2007	1.20 (0.86–1.66)	0.28
		GCTG	0.1395	0.1314	0.97 (0.67–1.40)	0.86
COL9A2	Block 1	GGCTGA	0.2963	0.3218	1.00	---
		GGCCGC	0.2614	0.2389	0.82 (0.59–1.15)	0.25
		GGTTTA	0.1502	0.1372	0.80 (0.53–1.20)	0.28
		GACTGA	0.1176	0.1114	0.83 (0.54–1.29)	0.42
		CGCCGC	0.0762	0.0891	1.08 (0.67–1.76)	0.74
		GGCCGA	0.0492	0.0375	0.66 (0.34–1.29)	0.22
		GGCTTA	0.0305	0.0370	1.00 (0.50–2.02)	0.99
COL9A3	Block 1	TT	0.5894	0.5500	1.00	---
		AT	0.2361	0.2817	1.29 (0.97–1.71)	0.086
		TC	0.1745	0.1683	1.06 (0.75–1.48)	0.74

### Association of polymorphisms of COL9A1 with the severity of KBD

It is not clear whether rs6910140 contributes to the severity of KBD. The association of rs6910140 in COL9A1 with the severity of KBD was monitored in the KBD group. Table 6 showed the distribution of alleles and genotypes for the subjects of the different KBD stages, the frequencies of genotypes and alleles in rs6910140 were significantly different among subjects of the three KBD stages. For the number of KBD patients with rs6910140 CC was very limited, dominant and recessive models were also applied to rectify this, the results showed an association of rs6910140 with the severity of KBD in dominant model (Table 6). Our findings showed rs6910140 in COL9A1 is related to the severity of KBD, and the wild type allele “C” has a significant decreasing effect on the severity of KBD.

**Table 6:** Comparison of genotype and allele frequencies of rs6910140 in COL9A1 among subjects of different KBD stages.

KBD stages	Alleles (%)		Genotypes (%)			Recessive model (%)		Dominant model (%)	
	T	C	TT	TC	CC	TT+TC	CC	TT	TC+CC
I(n = 158)	80.7	19.3	62.6	36.1	1.3	98.7	1.3	62.6	37.4
II(n = 85)	88.2	11.8	80.0	16.5	3.5	96.5	3.5	80.0	20.0
III(n = 31)	91.9	8.1	83.9	16.1	0.0	100.0	0.0	83.9	16.1
*X* ^2^ values	7.82		14.8			2.23		11.01	
*P* values	0.02		0.005			0.33		0.004	

## Discussion

The etiology of KBD remains unclear, genetic factors may particularly account for an individual’s susceptibility to KBD [6]. KBD and OA have similar pathological characteristics, such as chondrocyte necrosis and apoptosis, matrix degradation, and cartilage degeneration accompanied by absorption and repair [1], and mostly result in a secondary chronic osteoarthropathy [25]. The electron microscopic analysis have confirmed the chondrocyte necrosis and revealed a reduction in the collagen fibril diameter, and a loss of the fibril banding patterns in the cartilage matrix of the KBD patients [26].The role of type IX collagen in the degradative events invoked in the cartilage and bone of arthritic joints has long been appreciated. Several reports have suggested that the polymorphism of genes encoding collagen IX is associated with the risk of osteoarthropathy [14],[27]. However, no study has been conducted to investigate the relationship between KBD and COL9A. In this study, we selected twenty-seven SNPs from COL9A and performed an association analysis between COL9A and KBD.

In the present study, fifteen SNPs in COL9A1 were included to investigate the association between the gene COL9A1 and risk of KBD in the Chinese Han population. The distribution frequencies of genotypes in rs6910140 were significantly different between the two groups. The subjects carrying allele “C” have lower risk of KBD than the subjects without carrying allele “C”. Additional analysis showed that rs6910140 was significantly associated with the clinical diagnose of KBD stages. These results suggest that polymorphism of the COL9A1 plays an important role in the risk and severity of KBD in the Chinese Han population. Gene COL9A1 located on the long arm of chromosome 6. Several studies have provided evidences of association between COL9A1 gene and arthritis disease [16,28,29]. Our data also showed association between COL9A1 gene and KBD. Therefore, it is reasonable to infer that COL9A1 gene played an important role in one’s susceptibility to KBD who exposed to the same environmental. Locus rs6910140 is a non-synonymous SNP, there are limited studies for the function of rs6910140 in osteoarthropathy. Polymorphisms of rs6910140 in the COL9A1 gene may play an important role in determining the expression of a1(IX), we think it should be correlated to cartilage destruction by changing the expression of a1(IX) and related to KBD. Therefore, the α1(IX) protein expression and its effect on the risk of KBD should be explored in the following studies.

However, our findings did not support an association between the COL9A2, COL9A3 genes and KBD in our population, even though the heterozygosity of rs3765462 in gene COL9A3 showed a weak association with an evaluated KBD risk. However, the significance did not survive Bonferroni correction. Extensive studies have provided evidence for the associations between the genes COL9A2, COL9A3 and osteochondropathy [19,30,31]. For example, genetic study in a Finnish population identified two polymorphisms that predict the introduction of a tryptophan (Trp) residue into the a2 (IX) or a 3(IX) chain, the Trp-encoding alleles were linked to increased risk of lumbar disc disease [30,31]. An association study performed by Japanese group found an association between a SNP in COL9A3 and knee OA [19]. In contrast, the same SNP in COL9A3 was analyzed in the Finnish population, and no association was found [20]. Our own data suggested that the selected SNPs in COL9A2, COL9A3 genes were not major susceptibility genes in our studied population. However, we should not dismiss a possible association between the two genes and KBD. Our study was the first to explore the relationship between the polymorphisms of the selected genes and KBD, and there are also certain limitations in the study: The number of the selected SNPs was limited in genes COL9A2 and COL9A3 and the sample size was not large. Therefore the results need to be verified using well-designed, high-quality studies on well-defined populations.

In conclusion, we found that the rs6910140 in COL9A1 was associated with the susceptivity of KBD. The allele “C” of rs6910140 in COL9A1 had a significant protective effect on KBD. Further validation of the contribution of COL9A to the development of KBD in other populations is warranted.

## Supporting Information

S1 Dataset Information of genotypes of cases and controls. (XLS)
